# High gain quasi-omnidirectional dipole array fed by radial power divider for millimeter-wave IoT sensing

**DOI:** 10.1038/s41598-024-67032-7

**Published:** 2024-07-15

**Authors:** Md. Abu Sufian, Niamat Hussain, Domin Choi, Sang-Min Lee, Sang-Keun Gil, Nam Kim

**Affiliations:** 1 Department of Information and Communication Engineering, Chungbuk National University, Cheongju, 28644 Republic of Korea; 2 Department of Intelligent Mechatronics Engineering, Sejong University, Seoul, 05006 Republic of Korea; 3 Division of Creative Convergence, Korea National University of Transportation, Chungju, 27469 Republic of Korea; 4 Department of Electronic Engineering, The University of Suwon, Hwaseong, 445743 Republic of Korea

**Keywords:** Dipole array, mmWave antenna, Quasi-omnidirectional radiation, Radial waveguide power divider, Small antenna, Wireless sensing, Engineering, Electrical and electronic engineering

## Abstract

This article presents the design and implementation of a dipole array antenna based on a radial waveguide power divider for millimeter-wave IoT sensing applications. The dipole array and radial waveguide power divider techniques are used in tandem to achieve high gain with omnidirectional radiation properties. The proposed antenna is comprised of eight non-uniform array dipole structures, a circular radiating loop, and shorting vias. The one-to-eight power divider is created with the shorting vias to feed the circularly arranged eight non-uniform dipole arrays simultaneously. The proposed antenna is simulated and manufactured on Rogers-RO3003C substrate with a thickness of 8 mils. Both simulated and tested results confirm that the proposed method enables the antenna to offer a quasi-omnidirectional pattern with a high peak gain of 5.42 dBi. The antenna offers an impedance bandwidth (S_11_ < ‒ 10 dB) of more than 1 GHz ranging from 27.93 to 29.13 GHz. Moreover, by optimizing the parameters of the power divider network the proposed antenna can be tuned between a wide bandwidth range of 14.53 GHz as the designed dipole array offering the operating bandwidth from 25.56 to 40.09 GHz. Due to its comprehensive set of performance attributes, particularly for the quasi-omnidirectional radiation characteristics, the presented antenna is a viable candidate for the 5G millimeter wave wireless IoT sensing applications. Additionally, this work will accommodate other researchers to explore the proposed method for developing high-gain omnidirectional antennas for millimeter-wave applications.

## Introduction

The development of connected devices and data-driven gadgets has led to a remarkable growth in the adoption of the Internet of Things, revolutionizing several industries and daily life^1^. As a result of the Internet of Things' (IoT) accelerated and ongoing growth, billions of devices will be required to be connected to the Internet in the coming years in order to support applications like 5G mobile servers, Industry 4.0, advanced mobile communication, smart homes, and smart cities^2^. The Internet of Things (IoT) aims to establish a highly intelligent network that links all systems or devices to the Internet. Most of these gadgets are sensors that communicate with one another seamlessly and share data without the involvement of humans^3,4^. The IoT enables intelligent detection, recognition, and surveillance of objects across a range of real-time applications while sensing is one of the most crucial IoT features^5,6^. For the IoT sensing applications one of the possible solutions is the radio-frequency identification (RFID)^6–8^. However, the coverage of the RFID sensing is considerably limited as the RFID tags should be very close to the RFID reader to be sensed by the RFID reader^6,8^. Additionally, most of the RFID sensors are efficient for the kHz and MHz frequency bands^6–8^. For the millimeter-wave (GHz frequency bands) IoT sensing applications the antennas are more efficient and offer broader sensing coverage area.

**Figure 1:**
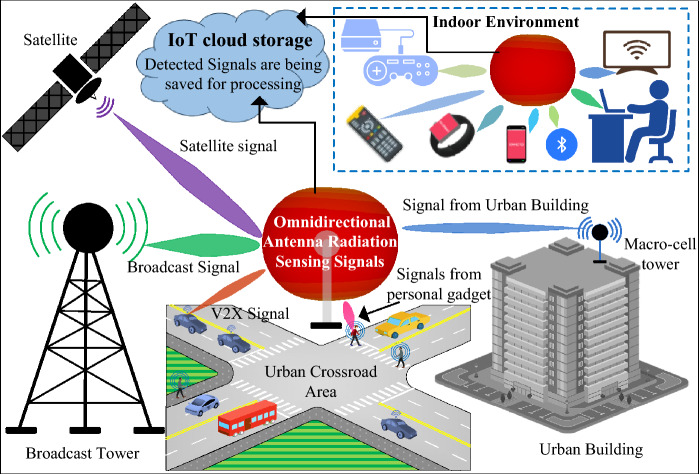
The widespread usage of omnidirectional antenna for the sensing applications.

The need for antennas is rising in tandem with the proliferation of IoT devices, particularly for 5G millimeter wave antennas, which offer higher internet speeds and wider channel capacity to support the exponential growth in connected devices^9,10^. Whereas the 28 GHz frequency band is the most widely used frequency band for millimeter-wave antennas worldwide^11,12^, and the majority of millimeter-wave wireless channels in South Korea have a bandwidth of 800 MHz^13^. For IoT applications, antennas with both unidirectional and omnidirectional radiation patterns are required based on their targeted uses^14–18^. Unidirectional antennas are superior to omnidirectional antennas when it comes to transmitting signals across larger distances as these antennas typically offer high gain^14,15^. On the other hand, when it comes to receiving signals from all directions, omnidirectional antennas outperform unidirectional antennas^16–20^. The widespread usage of omnidirectional antenna for the sensing applications are illustrated in Fig. 1.

In literature, a number of works have been reported for the millimeter-wave applications^21–32^, where various techniques have been utilized to improve the performance of the antennas for the millimeter-wave wireless communications including millimeter-wave IoT applications. In^21^, a slotted co-planar monopole antenna is designed to achieve high gain and omnidirectional radiation with multiband characteristics. The antenna in^21^ offers omnidirectional radiation in *H*-plane however on the *E*-plane the antenna shows elliptical radiation characteristics. In^22^, a slotted tapered antenna unified with a feeding network built on stacked PCBs is proposed to achieve high gain and unidirectional radiation characteristics for the millimeter-wave IoT applications. While, higher order resonant mode^23^, slotted radiating patch^24^, modified binomial series-fed array^25^, glass-based multilayer antenna integrated with coupling vias^26^, SIW metamaterial^27^, lens operated planar antenna^28^, tapered rectangular monopole with slotted ground^29^, artificial magnetic-conductor^30^, dielectric-resonator^31^, and radial-waveguided dipole array^32^ are investigated to achieve multi-band characteristics^23,24,29–31^, high gain^25,28^, process accuracy^26^, beam steering^27^, omnidirectional radiation patterns with high gain^29–32^. However, the majority of antennas described in the literature for millimeter-wave wireless communication, including Internet of Things applications, are primarily focused on high gain characteristics and provide unidirectional radiation patterns^22–28^. While the available omnidirectional antennas in the literature for millimeter wave applications are limited^29–32^, especially for IoT applications. Additionally, all these millimeter-wave omnidirectional antennas^29–32^ are suffer from low antenna gain. Alternatively, the dipole array approach is a particularly efficient way to boost the gain of millimeter-wave antennas; nevertheless, these works also provide unidirectional end-fire radiation characteristics^33–36^.

In this work, a new technique, non-uniform dipole array fed by a radial waveguide power divider is proposed and investigated experimentally to achieve quasi-omnidirectional radiation characteristics with high gain for millimeter-wave wireless IoT sensing applications. The proposed antenna consists of a circular radiating loop, cavity shorting vias, and eight non-uniform array dipole structures. The dipole arrays are positioned in a circular pattern with a 45° separation between them. By properly utilizing the shorting vias a one-to-eight power divider is created to feed the dipole arrays. The dipole array yields a higher gain than the other reported works, while the circular arrangement of the eight-dipole array offers quasi-omnidirectional radiation characteristics. The quasi-omnidirectional radiation pattern with the high peak gain of the presented work makes it a promising candidate for millimeter-wave IoT sensing systems. Based on the author's knowledge, till this manuscript is submitted, there is no reported millimeter-wave antenna in the literature with high gain and omnidirectional radiation characteristics for wireless IoT sensing applications. All the antenna simulation is computed in the CST Microwave Studio environment. The remaining sections of the manuscript are organized as follows. Section "Proposed antenna design methods" contains a detailed explanation of the antenna design methodologies, while the simulated and tested results of the proposed antenna are presented in Section "Antenna results"; followed by the performance comparison with the reported millimeter-wave antennas in the literature of Section "Performance analysis and comparison". Finally, the proposed work has been concluded in Section "Conclusion".

## Proposed antenna design methods

The design method of the proposed antenna is explained in this section. While, in the first subsection, the geometry of the proposed antenna is depicted with all its design parameters. Subsequently, the design procedure is explained.

### Antenna geometry

**Figure 2:**
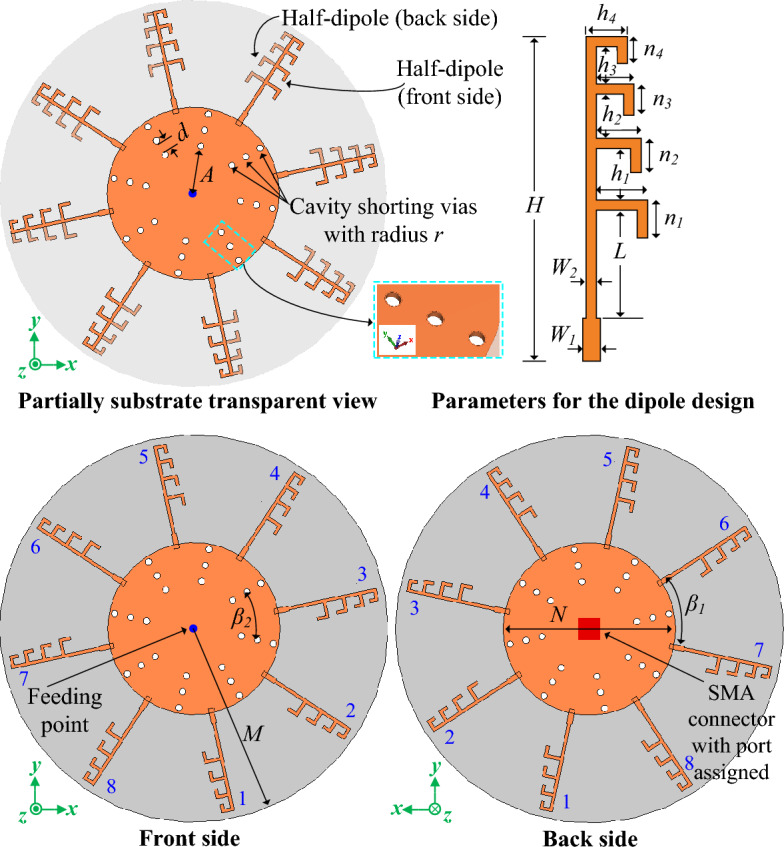
The antenna architecture in the simulation environment and the design variables of the proposed quasi-omnidirectional sensing antenna.

The schematic of the proposed dipole array antenna fed by radial waveguide power divider (RWPD), and the design variables of the proposed antenna are depicted in Fig. 2. The antenna is designed on Rogers-RO003C substrate with a thickness of 8 mils, while having the material attributes *ℇ*_*r*_ = 3.55, and *tanδ* = 0.0027. The antenna design methodology is described in the subsequent subsection. And the optimized parameters for the proposed antenna are listed in Table 1.

**Table 1:** The design parameters and optimized values.

Variable	Value	Variable	Value (mm)	Variable	Value (mm)
*M*	18 mm	*W*_*1*_	0.5	*h*_*3*_	1.1
*N*	16.5 mm	*W*_*2*_	0.3	*h*_*4*_	0.8
*r*	0.3 mm	*L*	3.2	*n*_*1*_	1.1
*A*	4.45 mm	*H*	9.2	*n*_*2*_	1.0
*β*_1_, *β*_2_	45°	*h*_*1*_	1.5	*n*_*3*_	0.9
*d*	0.9 mm	*h*_*2*_	1.3	*n*_*4*_	0.8

### Design procedure

In this sub-section, the design process of the proposed quasi-omnidirectional antenna with high gain is presented. At first, a millimeter-wave four-element non-uniform dipole array is designed, which offers an unidirectional end-fire radiation pattern with high gain. Afterward, the proposed one-to-eight radial waveguide power divider is designed for the 28 GHz frequency band. Subsequently, the dipole array and the power divider are combined together as shown in Fig. 2 to achieve omnidirectional radiation characteristics with high gain for the wireless sensing applications at 28 GHz millimeter-wave frequency band.

#### Design of the proposed millimeter-wave dipole array

The parameters of the designed dipole array are conveyed in Fig. 2. The total height of the dipole array is *H*, while the width of the dipole feedline is *W*_*1*_. Instead of a conventional uniform dipole array, for the proposed design we utilized a novel non-uniform dipole array. Whereas the distance between the arms and the length of the dipole array arms are non-uniform. However, in the conventional uniform dipole array, the distance between all arms, and the length of the arms are identical. The non-uniformity in distance and length of the proposed non-uniform dipole array for the *h* and *n* variables can be defined as Eqs. (1) and (2), respectively. Where the *h*_*i*_ and *n*_*i*_ are for the *i*^*th*^ number of the arm, and *∆h* and *∆n* are the specific reduction ratios of the dipole arm. In the proposed design the optimized values of the *∆h* and *∆n* are 0.2 mm and 0.1 mm, respectively. While the values of *∆h* = *∆n* = 0, the dipole array acts as the conventional uniform dipole array.

1 $$ h_{i} = \, h_{i - 1} {-}\Delta h $$

2 $$ n_{i} = \, n_{i - 1} {-}\Delta n $$

The total length of the first dipole arm is predicted using Eq. 3, where the total length of the first dipole arm is considered as the half wavelength of the targeted frequency of 28 GHz. In Eq. 3 the total length of the full dipole arm is 2 × (*h*_*1*_ + *n*_*1*_), while the (*h*_*1*_ + *n*_*1*_) is the length of the half dipole of the first arm. And λ is the wavelength of the targeted frequency. Subsequently the addition arms are designed by following Eqs. 1 and 2 which is stated above. Finally, the parameters are optimized for the optimal results. All the optimized parameter values are listed in Table 1.

3 $$2({h}_{1}+{n}_{1}) = \frac{\uplambda }{2}$$

**Figure 3:**
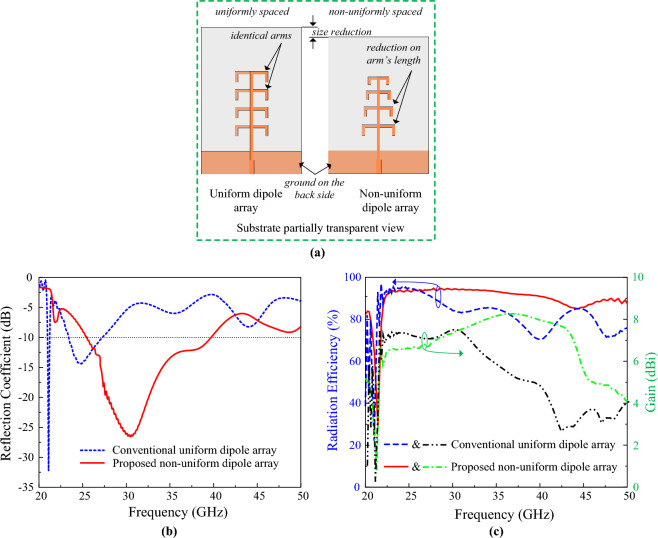
Comparative analysis of the conventional uniform dipole array and the proposed non-uniform dipole array (**a**) antenna geometry, (**b**) frequency response, and (**c**) radiation efficiency and gain response.

The designed non-uniform dipole array required less space than the conventional uniform dipole array, as the separation of two arms is continuously reduced in the proposed non-uniform dipole array as demonstrated in Fig. 3a. Moreover, the non-uniform dipole array offers a wider operating bandwidth than the conventional uniform dipole array. The impedance bandwidth performance of the proposed non-uniform dipole array and conventional uniform dipole array are presented in Fig. 3b. Additionally, it can be observed from Fig. 3c that the proposed non-uniform dipole array offers better radiation and gain performance than the conventional uniform dipole array.

**Figure 4:**
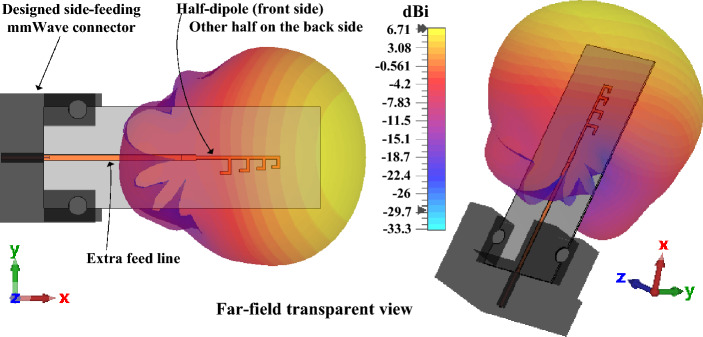
The radiation performance and gain of the designed non-uniform millimeter-wave dipole array antenna at 28 GHz.

The antennas in Fig. 3 are simulated using the ideal port connection available in the simulation environment. However, the millimeter-wave antennas are affected significantly while the antenna is connected (edge-feeding) with a real connector available in the market. To overcome the connector effect on the antenna’s performance study of the designed millimeter-wave dipole array antenna, the feedline of the antenna is additionally extended 13.6 mm, and an mmWave edge-feed connector is designed in the simulation software to get the actual performance as the manufactured antenna. The radiation performance and gain of the optimized non-uniform dipole array are presented in Fig. 4. For the proposed single element antenna four directors are considered as these number of director dipoles offer a moderate gain with a lower size. The increment of the directors will help to increase the gain at the same time it will increase the overall antenna size of the proposed antenna.

**Figure 5:**
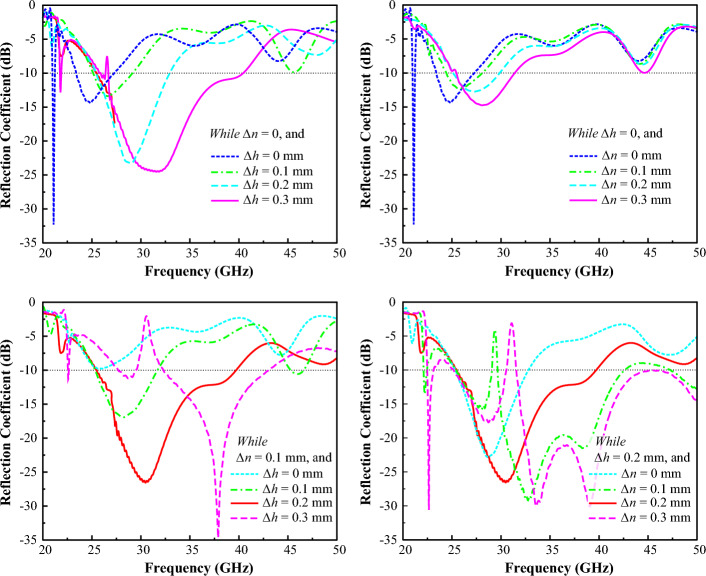
The parametric analysis of the designed non-uniform dipole array antenna for the antenna’s reflection coefficient at different values of *∆h* and *∆n*.

The parametric analysis of the designed non-uniform dipole array antenna is depicted in Fig. 5 for the antenna’s reflection coefficient at different values of *∆h* and *∆n*. It can be observed from the parametric study that the impedance bandwidth of the designed non-uniform dipole array can be controlled by varying the values of *∆h* and *∆n*. It is also found out that with the increment of the *∆h* and *∆n,* which results in the more continuous reduction on the length of the dipole arms and the distance between the dipole arms, the operating impedance bandwidth is shifting to the upper band. However, by controlling the *∆h* and *∆n* the frequency cannot be shifted to the lower band. Therefore, the initial uniform dipole array should be designed for the expected lower band and afterward by varying the *∆h* and *∆n* the operating bandwidth of the dipole array can be increased toward the upper frequency band.

#### Proposed RWPD fed dipole array antenna

The proposed millimeter-wave non-uniform dipole array is designed and optimized for a wide operating bandwidth of 14.53 GHz (from 25.56 to 40.09 GHz) with an average gain of more than 6.5 dBi within the operating frequency band. As the dipole array alone offers an end-fire unidirectional radiation characteristic, a one-to-eight radial waveguide power divider is designed to feed eight dipole arrays simultaneously using a single feeding network to achieve an omnidirectional radiation pattern with high gain.

Additionally, as the designed dipole array network is capable of operating between 25.56 and 40.09 GHz. By optimizing the power divider network the antenna can be operated within this broader frequency spectrum. However, the majority of millimeter-wave systems operate on an 800 MHz bandwidth channel worldwide including in South Korea^13,37^. While the 28 GHz band (n257) is mostly used for millimeter-wave communication by most of the countries all over the world^13,37,38^. For this reason, in this work, the power divider network is designed and optimized for an 800 MHz bandwidth at the 28 GHz frequency band. The allocated channels at the 28 GHz frequency band in South Korea and the corresponding frequency range are listed in Table 2.

**Table 2:** Allocated channels at 28 GHz band in South Korea.

Total Bandwidth	Channels	BW (MHz)	Frequency range (GHz)
2400 MHz (26.5–28.9 GHz)	Channel-1	800	26.5–27.3
	Channel-2	800	27.3–28.1
	Channel-3	800	28.1–28.9

**Figure 6:**
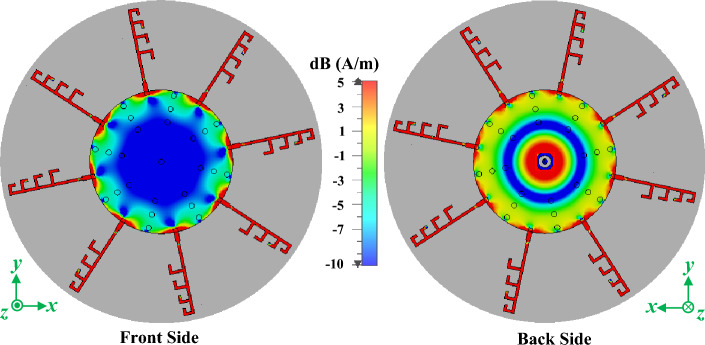
The surface current distribution of the proposed RWPD-fed dipole array antenna at 28 GHz.

The proposed RWPD-fed dipole array antenna consists of an SMA connector, a circular radiating loop, cavity shorting vias, and eight non-uniform four-element array dipole structures as illustrated in Fig. 2. With a diameter of *N*, the radiating circular loop is formed on both the front and rear sides and is fed via the SMA connector. To supply power to the eight distinct dipole structures, a one-to-eight radial waveguide power divider is created by the three sets of eight cavity shorting vias, each having a radius of *r*. The initial group of shorting vias is positioned at a distance of *A* from the center of the entire geometry. In every set of shorting vias, there is an angle of *β*_*2*_ between two adjacent shorting vias. And between the two closest sets of shorting vias, there is a separation of *d*. By optimizing these parameters, the power divider is tuned for the 28 GHz frequency band. Thanks to CST Microwave Studio's translate, copy, and rotate tools, this complex layout is implemented with ease. Afterward, the designed dipole structure is positioned between two lines of the cavity shorting vias as depicted in Fig. 2, while half of the dipole is placed on the front side and another half on the backside. Finally, the designed dipole is translated and copied with a replication factor of seven and an angle of *β*_*1*_ to create a total of eight dipole structures in the CST simulation environment. Figure 2 defines the parameters needed to design the dipole structure. After the design is accomplished, the parameters are tuned for the best performance by targeting the 28 GHz frequency band with a bandwidth of 800 MHz (from 28.1 to 28.9 GHz). From the surface current distribution at 28 GHz of the proposed antenna in Fig. 6, it can be realized that the optimized power divider works perfectly at the 28 GHz frequency band.

**Figure 7:**
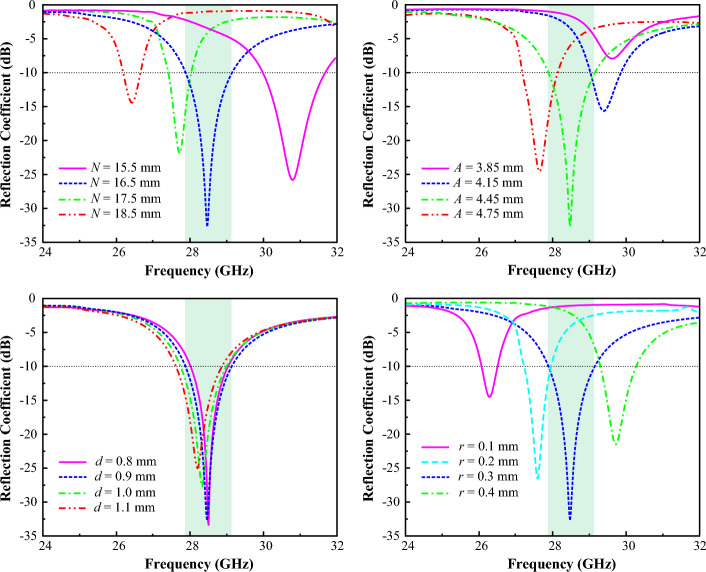
Response of various parameters at different values of the power divider network of the proposed RWPD-fed dipole array antenna.

The parametric study of the power divider network for feeding the power to the dipole array network is presented in Fig. 7, and the corresponding parameter can be realized from Fig. 2. It can be seen from Fig. 7 that the parameters *N* and *r* play a significant role to control the frequency response for the proposed antenna. While with the increment of the value of *N,* the antenna resonance is shifting from the upper band to the lower band. And the operating frequency shifts from the lower band to the upper band as a result of the increment of the value of the radius of the cavity vias *r*. On the other hand, by optimizing the parameters *A* and* d* the quality of the resonance can be improved. The optimized parameters of the proposed radial waveguide power divider-based dipole array antenna for the results presented in this paper are listed in Table 1.

## Antenna results

**Figure 8:**
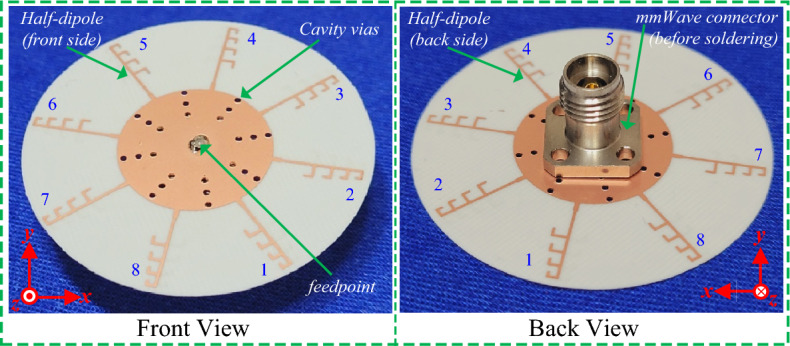
Fabricated prototype photograph of the proposed antenna.

**Figure 9:**
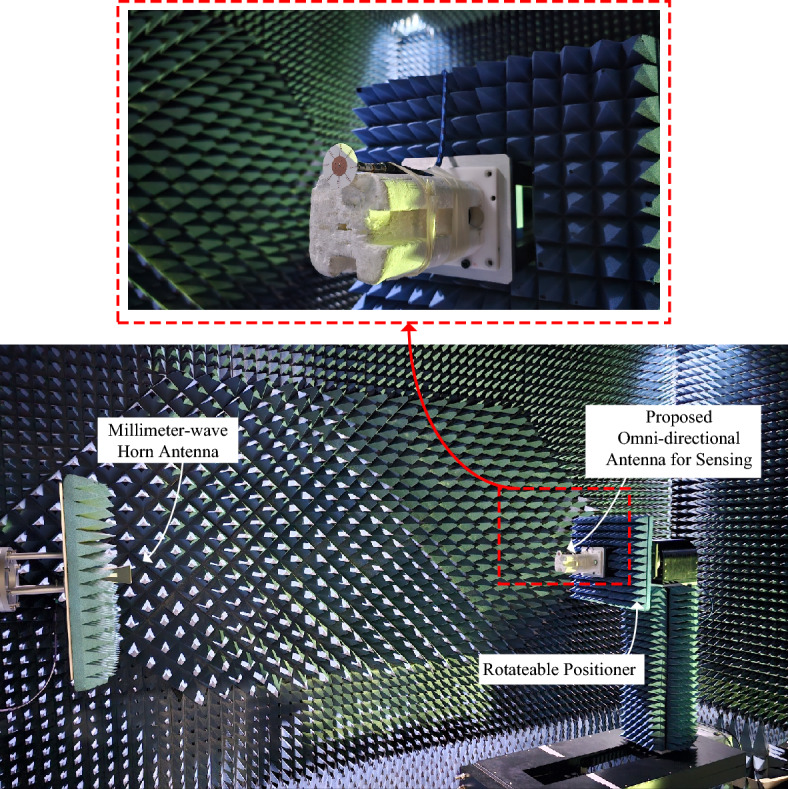
Far-field measurement setup of the proposed antenna in the millimeter-wave anechoic measurement chamber.

In the section, the findings, both simulation and measurement, of the proposed antenna are presented. Both the measurement and simulation findings validated the proposed work. The proposed antenna, dipole array fed by an RWPD is manufactured, and tested. The fabricated antenna prototype is shown in Fig. 8, while the antenna photograph in the anechoic chamber for the far field characteristics measurement setup is presented in Fig. 9. At the measurement chamber a millimeter-wave horn antenna is used to send the signal, while the proposed quasi-omnidirectional antenna receives the signal.

### Reflection coefficient

**Figure 10:**
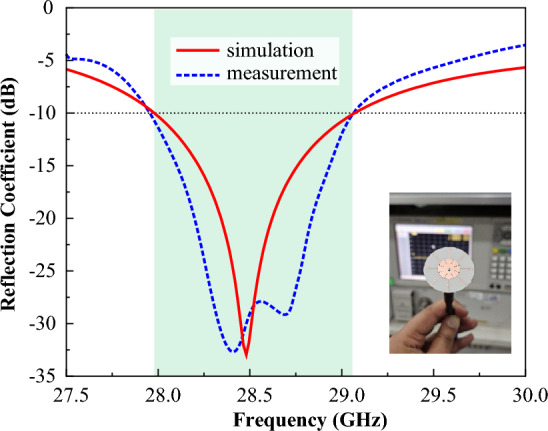
Reflection coefficient response of the proposed radial waveguide-based dipole array antenna.

The reflection coefficient response of the proposed RWPD-fed dipole array antenna is displayed in Fig. 10, while the reflection coefficient is analyzed in the open-air environment by means of the Agilent E83664B network analyzer. Due to the measurement equipment losses and the connector losses, there is little variation between the simulated and tested findings. The antenna is targeted to cover the mostly used 28 GHz channel of 800 MHz (from 28.1 to 28.9 GHz) in South Korea^13,39^. The presented antenna provides a (S11 < − 10 dB) bandwidth of more than 1000 MHz (27.93–29.13 GHz), covering the entire targeted frequency band. Therefore, the proposed antenna can sense any signal in the range between 27.93 and 29.13 GHz.

### Radiation pattern

**Figure 11:**
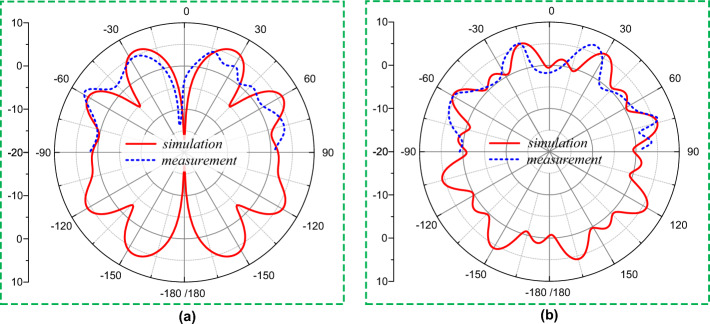
2D-polar radiation characteristics of the proposed RWPD-fed dipole array antenna at 28.5 GHz (**a**) elevation plane, and (**b**) azimuth plane.

**Figure 12:**
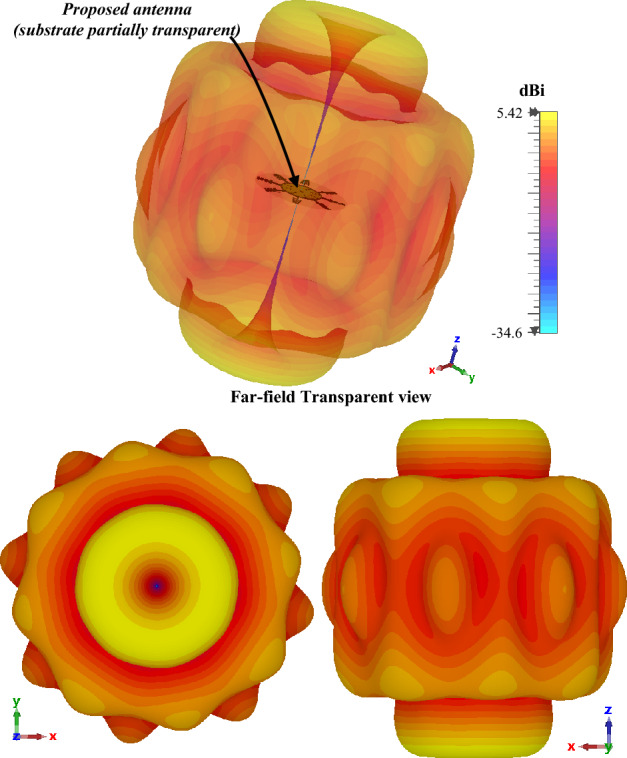
3D radiation pattern of the proposed antenna at 28.5 GHz.

The computed and tested 2D-polar radiation pattern of the proposed power divider fed dipole array antenna is demonstrated in Fig. 11 at 28.5 GHz for both elevation and azimuth plane. While the 3D radiation pattern of the proposed antenna at 28.5 GHz is simulation software is presented in Fig. 12. The radiation characteristics of the proposed antenna are measured by a commercial antenna research facility in an anechoic chamber as shown in Fig. 9^40^. This millimeter-wave chamber^40^ is capable of measuring the antenna radiation from + 90° to − 90° by rotating the positioner at both elevation and azimuth plane. The measurement radiation pattern shows a close similarity with the simulated results in the measured range of + 90° to − 90°. As the dipole array structure are spaced uniformly in the proposed antenna, it can be realized that at the unmeasured direction (+ 90° → 180°/− 180° → − 90°) it will also offer the identical radiation characteristics.

From both the 2D-polar and 3D radiation pattern of the presented antenna from Figs. 11 and 12 respectively it can be observed that the antenna yields an quasi-omnidirectional radiation pattern. The quasi-omnidirectional pattern of the proposed antenna enables it to act as a wireless sensor for the IoT sensing applications, which will sense signals from all directions with an average gain of higher than 4 dBi. The antenna offers a similar radiation characteristic within the functional frequency range. The omnidirectional radiation characteristics of the presented antenna make it a good fit for wireless sensing applications.

### Antenna efficiency

**Figure 13:**
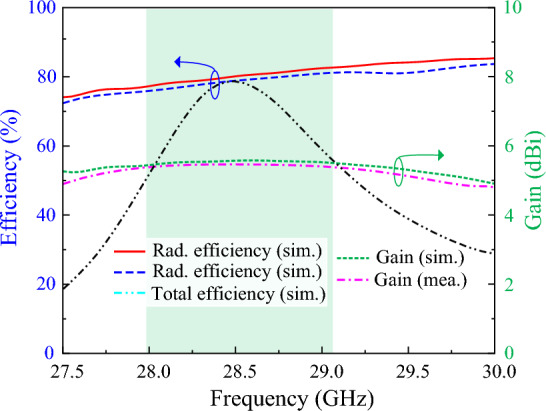
Total efficiency and gain of the proposed RWPD-fed dipole array antenna.

The radiation efficiency of the manufactured antenna is displayed in Fig. 13 for both the simulation and measurement. Radiation efficiency shows how efficiently an antenna can send or receive RF signals. From both simulated and tested findings, it can be seen that the antenna offers a radiation efficiency between 78 and 83% within the functional frequency range. The tested radiation efficiency level is little mitigated than the simulated radiation efficiency due to the losses of the different equipment during the measurement. The radiation efficiency of the proposed antenna ensures its capability for the signal receiving. Additionally, the simulated total efficiency of the proposed antenna is also presented in Fig. 13. The antenna offers a peak total efficiency of 79% at 28.5 GHz, while the suggested antenna maintains a total efficiency of more than 50% within the functional frequency band with an average total efficiency of more than 65%.

### Gain

The gain of the proposed antenna is also exhibited in Fig. 13. The proposed antenna offers a peak gain of 5.42 dBi at 28.5 GHz with an average stable gain of higher than 4dBi within the functional frequency band (27.93–29.13 GHz). By optimizing the power divider network and utilizing the dipole array structure a high gain is achieved while providing quasi-omnidirectional radiation characteristics.

## Performance analysis and comparison

Table 3 conveys a performance comparison of the previously reported millimeter-wave antenna and the proposed RWPD-fed dipole array millimeter-wave antenna. The presented antenna yields a high peak gain of 5.42 dBi, while maintaining an omnidirectional radiation pattern characteristic. Moreover, the proposed antenna offers a high radiation efficiency of 83%. The comparison is done in terms of antenna size, antenna profile, radiation efficiency, peak gain, and the radiation type of the antenna. Whereas λ is the wavelength at the lower operating frequency.

**Table 3:** Performance comparison of the proposed antenna with existing millimeter-wave antennas.

References	Antenna size (mm^2^)	Profile (mm)	Radiation efficiency	Peak gain (dBi)	Radiation type
^22^	518.36 λ	0.508	80%	14.7	Unidirectional (End-fire)
^23^	36.02 λ	1.32	60%	7.7	Unidirectional
^27^	373.66 λ	1.57	Not given	15	Unidirectional
^29^	14.94 λ	0.38	78%	1.27	Omnidirectional
^30^	14.65 λ	0.254	82%	4.86	Omnidirectional
^31^	14.60 λ	2.01	90%	4.3	Omnidirectional
^32^	26.21 λ	0.508	85%	1.98	Omnidirectional
Prop	94.83 λ	0.203	83%	5.42	Omnidirectional

It can be seen from the comparison table that the antennas with unidirectional radiation patterns can offer a high peak gain^22,23,27^. However, for the wireless signal sensing applications the antennas with unidirectional radiation characteristics are not suitable. Additionally, these antennas either have a larger antenna size^22,27^, or operate with a very low radiation efficiency^23^. Other the other hand, the reported antennas with omnidirectional radiation pattern^29–32^ offer very low antenna gain, while the antenna^29^, and^32^ have a gain value of less than 2dBi. A high radiation efficiency is offered by the antenna reported in^31^, however, the antenna suffers from the higher antenna profile. Among the antennas with omnidirectional radiation characteristics^29–32^, the proposed antenna offers a higher gain which ensures better wireless sensing capabilities. The overall performance of the proposed RWPD-fed dipole array antenna makes it a strong candidate for millimeter-wave wireless IoT sensing applications.

## Conclusion

In this paper, a dipole array fed by a radial waveguide power divider for omnidirectional radiation characteristics with high gain is developed and implemented for the 28 GHz band millimeter-wave wireless IoT sensing applications. The combination of the dipole array with the designed power divider network offers a high gain with quasi-omnidirectional radiation. Initially, a four-element non-uniform dipole array is designed for the 28 GHz millimeter-wave band. While a set of equations is also developed in this manuscript to design the non-uniform dipole array. The optimized dipole array antenna offers unidirectional end-fire radiation with a peak gain of 6.71 dBi. And the dipole array network is designed to operate between 25.56 and 40.09 GHz. Subsequently, the power divider network is designed to feed eight dipole arrays, and the dipole array network is integrated with the power divider network by copied and rotated with the factor of seven and 45°, respectively. The power divider network consists of a circular patch and cavity shorting vias. By controlling the size of the circular patch, and the positions and gaps between the vias the operating frequency of the proposed antenna can be optimized between 25.56 and 40.09 GHz, as the dipole array network is designed for this frequency range. However, the authors are focused on the 28 GHz band with an 800 MHz bandwidth. The proposed antenna is fabricated on a circular-shaped Rogers-RO3003C substrate with a thickness of 8 mils. The antenna occupies a compact size of 94.83 λ, whereas λ is the wavelength at the lower operating frequency. Both simulated and measured findings indicate that the finalized antenna offers a S_11_ < − 10 dB impedance bandwidth of more than 1 GHz ranging from 27.93GHz to 29.13, which covers the targeted channel bandwidth of 800 MHz (28.1–28.9 GHz). Moreover, the antenna offers an omnidirectional radiation with a peak gain of 5.42 dBi, and radiation efficiency more than 78% within the functional frequency range. The overall performance metrics of the presented antenna make it a capable and adequate candidate for the 28 GHz band wireless IoT sensing applications.

## Data Availability

The presented paper contains all the data required for evaluating the findings of this research. The corresponding author can be contacted for more data regarding this work.
